# How Do Pediatricians Diagnose Asthma in Tertiary Care Hospitals?

**DOI:** 10.7759/cureus.29768

**Published:** 2022-09-30

**Authors:** Ghulam Mustafa

**Affiliations:** 1 Pediatric Medicine, College of Medicine Shaqra University, Shaqra, SAU; 2 Pediatric Medicine, Nishtar Medical University, Multan, PAK

**Keywords:** asthma diagnosis, audit, feno, spirometry, peak flow meter, pediatrician, children, asthma

## Abstract

Objective: There is a lot of disparity in the guidelines and the practice of pediatricians globally for diagnosing asthma in children. To find out if pediatricians are diagnosing asthma in children according to best standard practices.

Methodology: A cross-sectional study was conducted at tertiary care hospitals' emergency and outpatient departments (OPDs). All the parents accompanying the asthmatic children to the emergency or outpatient departments of the tertiary care hospitals were asked questions regarding the diagnosis of their children's asthma on a prescribed performa. This performa had all the components of the best standard practices for the diagnosis of asthma in children. The data were entered into SPSS version 27 (SPSS Inc., Chicago, IL) and analyzed.

Results: Among the 234 children, the diagnosis of asthma was based on only one component out of three, i.e., recurrence (100%) of symptoms or signs. The objective measurement of the second component, i.e., reversibility with a peak flow meter (PFM) or spirometry, was assessed in only 6% of children. The third component, i.e., the presence of inflammation, was not assessed at all (0.0%).

Conclusions: The diagnosis of asthma in children lacks precision. This is far from the evidence-based best standard practices. There is a need to provide motivation, training, and equipment to the staff.

## Introduction

Asthma is the commonest chronic inflammatory disorder of the lungs with heterogenous symptoms and signs, so it is no wonder that it is variably defined in various guidelines around the world [1-7]. But all these guidelines concur on three components of the asthma definition, i.e., recurrence of symptoms, airflow obstruction with evidence of reversibility, and the presence of chronic inflammation. The majority of the guidelines have been emphasizing the first two components for the diagnosis of asthma, partly due to the non-availability/cost of equipment to measure chronic inflammation [8-10]. However, they have been stressing that airflow obstruction and reversibility should not only be observed subjectively but also be objectively measured with peak flow meter (PFM), bronchodilator reversibility, bronchial challenge testing, or obstructive spirometry [1,4,5]. However, more recent guidelines have started recommending the need for the presence of chronic inflammation in the airway for the diagnosis of asthma by measuring fractional excretion of nitric oxide (FeNO) in the expiratory flow [9,11,12].

Our study aimed to find out how doctors diagnose asthma in children at tertiary care centers in comparison with the guidelines recommended, evidence-based, best standard practices.

## Materials and methods

The study was conducted at the emergency and outpatient departments (OPDs) of the Children's Hospital and the Institute of Child Health (CH & ICH), Multan & the Nishtar Medical University and Hospital (NMU & H), Multan. All the children, between the age of 4 and 18 years, who came to the emergency and OPDs for the first time with a prior diagnosis of asthma were included, except the children who came for follow-up.

The sampling technique was convenient sampling, and the sample size was calculated by OpenEpi version 3.0. The population size (75,000) was based on the 5% asthma prevalence in our population of 1.5 million. Keeping the confidence level at 95%, the likely positive outcome factor at 15%, and the margin of error at 5%, the sample size was estimated as 196.

The attending doctor asked a few questions from the parents (who consented) regarding the way their child had been diagnosed as having asthma. The responses were recorded on a specified proforma. The performa was prepared based on the very definition of asthma, i.e., the recurrence of episodes, the reversibility of bronchoconstriction with or without pharmacotherapy, and chronic inflammation. For the recurrence of symptoms, we asked about the presence of afebrile breathlessness accompanied by wheeze (heard by the parents or doctor), nocturnal cough, exercise-induced cough, seasonal exacerbations, triggered induced cough, personal atopy, and family history of atopy or asthma. The broncho-constriction and reversibility were evaluated based on subjective and objective measurements (using a peak flow meter or spirometry). We asked if the bronchoconstriction was relieved with oral or inhaled bronchodilators or steroids. For chronic inflammation of the airways, we asked if the child had been assessed for inflammatory markers like fractional excretion of nitric oxide (FeNO) in the expiratory breath.

The study was approved by the Institutional Review Board (IRB) of the Nishtar Medical University, Multan, via letter number 15501 dated August 7, 2021. The data was entered in SPSS-27 and analyzed by measuring frequencies of various parameters against the evidence-based recommendations for the diagnosis of asthma in children.

## Results

**Figure 1:**
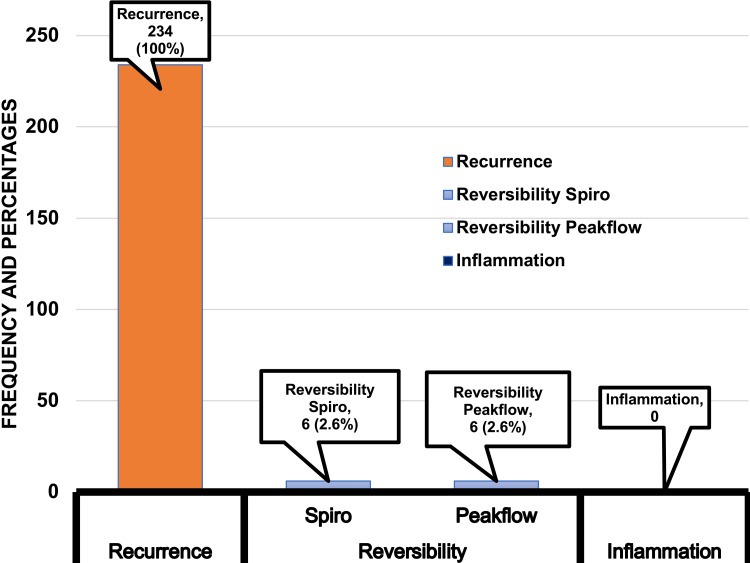
Asthma diagnosis components elicited by doctors

Of the 234 children, 2/3 (67.5%) were males. The majority (70.9%) of children were in the 5-12-year age group and presented with OPD (78.6%). The recurrence of symptoms was seen in 100% of children, which was noticed by the parents 98 (41.9%), doctors 80 (34.2%), or both 56 (23.9%). The reversibility of symptoms or bronchoconstriction was noted merely subjectively, with or without pharmacotherapy (Figure 1).

**Figure 2:**
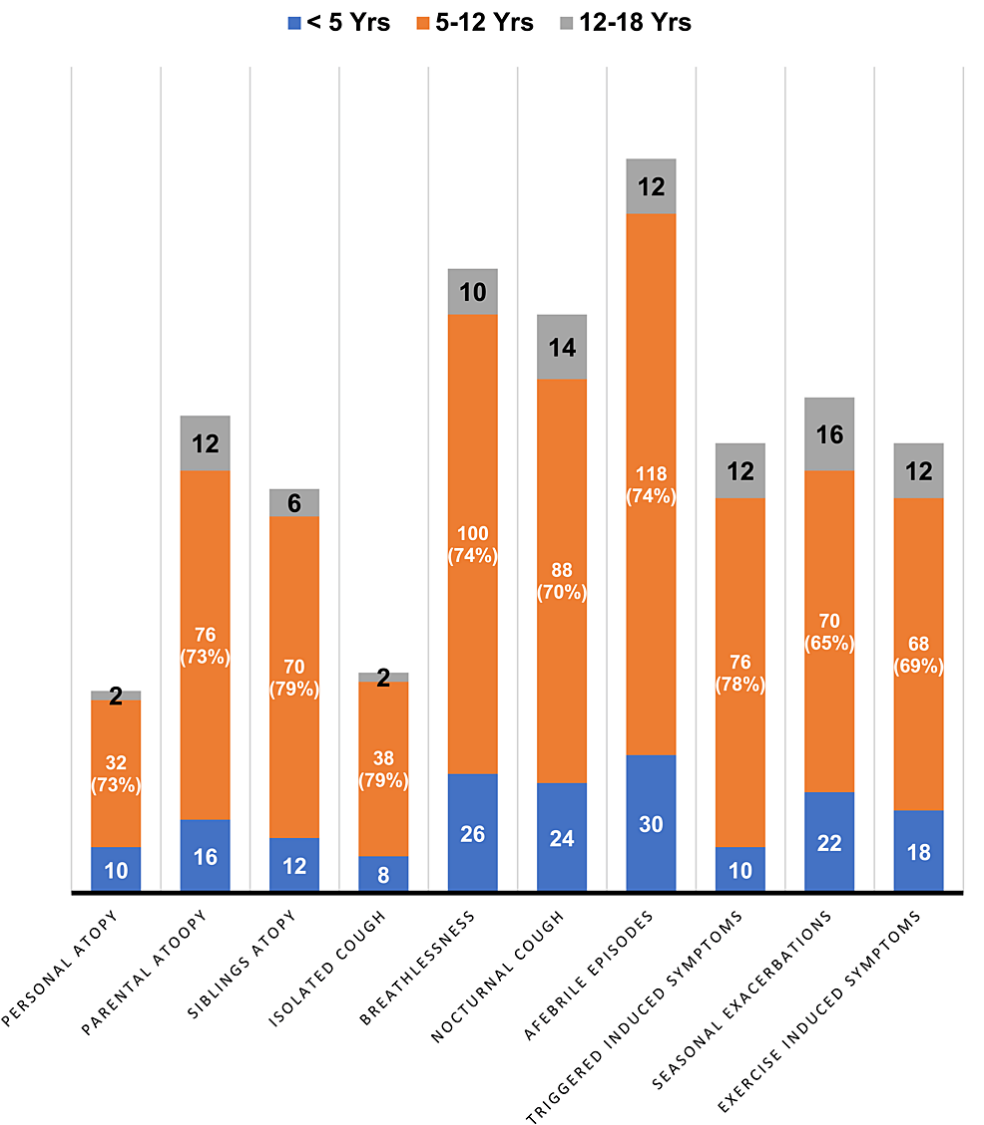
Asthma diagnosis recurrent history features in children

The objective measurement of bronchoconstriction or reversibility was carried out in barely 6 (2.6%) children with peak flow meter and spirometry (Figure 2).

FeNO (chronic inflammation) was not measured in any child. The frequency of symptoms, atopy in child/parent, and asthma in father/mother/siblings are shown in Table 1.

**Table 1:** Characteristics of children with asthma

(N = 234)	n (%)
Male	158 (67.5)
Female	76 (32.5)
Less than 5 years	44 (18.8%)
Male	38 (86.4)
Female	6 (13.6)
5-12 years	166 (70.9%)
Male	108 (65.1)
Female	58 (34.9)
12-18 years	24 (10.3%)
Male	12 (50)
Female	12 (50)
Mode of presentation	
Emergency	50 (21.4)
OPD	184 (78.6)
Asthma in parents	
Mother	56 (23.9)
Father	40 (17.1)
Both	10 (4.3)
Asthma/atopy in siblings	
Present	88 (37.6)
1 sibling	56 (23.9)
2 siblings	20 (8.5)
3 siblings	12 (5.1)
(N = 234)	n (%)
Male	158 (67.5)
Female	76 (32.5)
Less than 5 years	44 (18.8%)
Male	38 (86.4)
Female	6 (13.6)
5-12 years	166 (70.9%)
Male	108 (65.1)
Female	58 (34.9)
12-18 years	24 (10.3%)
Male	12 (50)
Female	12 (50)
Mode of presentation	
Emergency	50 (21.4)
OPD	184 (78.6)
Asthma in parents	
Mother	56 (23.9)
Father	40 (17.1)
Both	10 (4.3)
Asthma/atopy in siblings	
Present	88 (37.6)
1 sibling	56 (23.9)
2 siblings	20 (8.5)
3 siblings	12 (5.1)

## Discussion

Though asthma is defined variably by one of the biggest authorities, i.e., the European Respiratory Society (ERS) defines it as "Asthma is a disease that includes the symptoms of wheeze, cough, and breathing difficulty together with reversible airway obstruction, airway inflammation, and bronchial hyper-responsiveness" [11]. Almost the same is the wording of the American Academy of Pediatrics’ endorsed NAEPP (National Asthma Education and Prevention Program) guidelines that define asthma as "a common chronic disorder of the airways that is complex and characterized by variable and recurring symptoms, airflow obstruction, bronchial hyper-responsiveness, and an underlying inflammation" [13]. Keeping definitions like these in mind, various guidelines all over the world have recommended best-standard practices for the diagnosis of asthma in children aged 5-16 years [6,11,12,14,15].

The first best standard of practice for the diagnosis of asthma is the documentation of the recurrence of respiratory symptoms and signs. These symptoms and signs include, but are not limited to, isolated cough, nocturnal cough, exercise-induced cough or respiratory distress, seasonal cough or respiratory difficulty, chest tightness, and wheezing. The presence of asthma or atopic manifestations in the child, siblings, or parents adds to the diagnostic certainty [2,8,11,12,14].

The second best standard of practice for the diagnosis of asthma is the presence of reversible airway/airflow obstruction. The reversibility of the bronchoconstriction may be with or without pharmacotherapy. The best standard practice is to document this reversibility objectively [1,2,5,9,12,16,17]. The objective methods include spirometry, two-week monitoring of peak expiratory flow rate (PEFR), and bronchial challenge testing [11]. A few low-resource countries, due to the widespread non-availability of the equipment and non-affordability of the patients, have been reluctant to add objective documentation of the reversibility of bronchoconstriction for diagnosing asthma. They relied on the subjective improvement in the symptomatic bronchoconstriction as reported by the patients [3,7,18]. With the widespread availability of spirometry and low-cost peak flow meter, almost all asthma management guidelines recommend objective measurement of the reversibility of the bronchoconstriction to diagnose asthmatic children [16].

The third best standard of practice for the diagnosis of asthma is to document the presence of chronic inflammation in the airway as measured by the level of FeNO in the expired air [11,12,14]. With more and more availability of equipment around the globe, more and more guidelines are recommending the use of this modality for asthma diagnosis [6,8,11,12,14].

The latest UK National Institute of Health and Care Excellence guidelines recommend the use of spirometry, bronchodilator reversibility, and FeNO assessment as the first-line diagnostic tests for children with suspicion of asthma [12]. It argues that introducing these tests into all healthcare facilities will reduce misdiagnosis, improve asthma management, and reduce the cost of asthma care.

On the other hand, a hard fact is that there is a large gap between the recommended best-standard practices and the practices of pediatricians or physicians, all over the globe, for the diagnosis of asthma in children. Pre-hospital emergency care providers in South Africa used peak meters in only 8% of patients to manage acute asthma [19]. A study involving 816 internal medicine doctors, general physicians, chest physicians, and pediatricians from five countries (Morocco, Lebanon, Nepal, Malaysia, and Myanmar), showed that only 38% of them always used spirometry for diagnosing asthma [20]. In a national survey of primary care clinicians/physicians in America, the use of spirometry was reported to range from 6.8% to 16.8% at various centers [21]. In another nation-wide survey of American pulmonologists and allergists, only 36.6% and 56.6%, respectively, were using spirometry for diagnosing asthma in their patients [22]. In a UK study, 54% of children, who reported good asthma control, had abnormal spirometry and/or FeNO. It concluded that a symptom-based approach, without objective testing, to tackle asthma will likely miss children who are at high risk of severe asthma attacks [23].

The situation is not different in Pakistan. A nation-wide survey, involving 15000 households from seven major cities (almost one-third were children), showed that only 15% of the patients had lung function tests for the diagnosis of asthma [24]. This number includes children and adults and therefore mainly represents adult patients.

Our results are quite similar to the other studies from the region, like Myanmar, where just 6% of the healthcare providers always used spirometry for the diagnosis of asthma [20]. Our study shows that the diagnosis of asthma in our children is based on only the recurrence of the symptoms/signs and the subjective assessment of the reversibility of the bronchoconstriction. The objective measurement with spirometry or measurement of FeNO in expired air is not done as recommended by the latest guidelines. To conclude, the diagnosis of asthma is not optimal in our children as compared to the evidence-based best standard practices. This, inevitably, leads to the under-diagnosis and wrong classification of asthma severity. Both these facts entail inappropriate treatment, inapt loss of hospital resources, and financial loss of patients for managing this chronic disorder of the children.

Limitations

We did not look into the factors associated with the non-compliance of the doctors with the evidence-based best standard practices. Also, we only included children from the public sector hospitals and not from the private hospitals of the region, though that is not likely to affect the results. Because private sector hospitals are not more resourceful in having gadgets for the measurement of chronic inflammation or bronchoconstriction of the airways.

## Conclusions

There is a dire need to programmatically train doctors regarding the best standard practices regarding the diagnosis of asthma in children aged 5-16 years. They also need to be motivated to follow the guidelines to avoid underdiagnosis and inappropriate classification of asthma. An important aspect is to provide peak flow meters and spirometry equipment at all healthcare facilities.
